# Acute liver failure as the initial presentation of Wilson’s disease in a young adult: a case report

**DOI:** 10.1097/MS9.0000000000005551

**Published:** 2026-08-20

**Authors:** Sarbottam Koirala, Susant Thapa, Susmita Khadka Chhetri

**Affiliations:** Department of Pulmonary, Critical Care and Sleep Medicine, BPKIHS, Dharan, Nepal

**Keywords:** acute liver failure, hepatic encephalopathy, liver transplant, Wilson’s disease, young adult

## Abstract

Acute liver failure is a rapidly progressive condition with high mortality that requires early identification and timely intervention. Wilson’s disease is a rare but important cause of acute liver failure in young individuals. We report a 31-year-old, previously healthy man who presented with acute jaundice and progressively altered sensorium and was found to have grade III hepatic encephalopathy with markedly elevated transaminases, hyperbilirubinemia, and severe coagulopathy. An extensive evaluation excluded viral, autoimmune, and drug-induced causes. Further workup revealed low serum ceruloplasmin levels and Kayser–Fleischer rings, supporting a diagnosis of Wilson’s disease. Despite intensive supportive management, the patient showed biochemical deterioration with declining transaminases and worsening synthetic dysfunction, indicating progressive hepatic necrosis. He was referred for urgent liver transplantation; however, due to financial constraints, definitive treatment could not be pursued, and the patient was lost to follow-up. This case highlights the critical impact of socioeconomic barriers on access to life-saving therapy and clinical outcomes.

## Introduction

Wilson’s disease is an autosomal recessive disorder caused by mutations in the *ATP7B* gene, resulting in impaired biliary copper excretion and progressive copper accumulation in hepatocytes. This leads to ongoing hepatocellular injury and, in advanced stages, the systemic deposition of copper affecting multiple organs, including the liver, brain, kidneys, and musculoskeletal system, manifesting as hepatic, neurological, psychiatric, and extrapyramidal features^[^1,2^]^.

Early recognition of Wilson’s disease in patients presenting with acute liver failure (ALF) is crucial, particularly in young, non-alcoholic individuals after exclusion of infectious, toxic, and drug-induced causes. High clinical suspicion, supported by appropriate biochemical and ophthalmological findings, is essential for timely diagnosis^[^3,4^]^.

## Case summary

A 31-year-old previously healthy male presented with a 1-week history of recurrent vomiting, followed by an acute onset of jaundice and a progressively worsening altered sensorium. There was no prior history of liver disease, alcohol use, hepatotoxic drug intake, herbal medication use, or toxin exposure.

HIGHLIGHTS

- Acute liver failure in a young adult with rapid progression to grade III hepatic encephalopathy and severe coagulopathy.
- We ruled out common causes, such as infectious and drug-induced causes.
- Wilson’s disease was identified as the underlying cause based on low ceruloplasmin levels, the presence of Kayser–Fleischer rings, and a Leipzig score of 5, after exclusion of common etiologies.
- The Dhawan prognostic index for Wilson’s disease is 12, highlighting the urgent need for liver transplantation as the definitive management.
- A poor socioeconomic background hindered definitive management.

On admission, the patient was markedly icteric and exhibited altered mental status consistent with grade III hepatic encephalopathy, characterized by disorientation and incoherent verbal responses. Vital signs were stable. There were no stigmata of chronic liver disease. Abdominal examination revealed no hepatosplenomegaly or ascites.

Initial laboratory evaluation was consistent with ALF, demonstrating severe hepatocellular injury and coagulopathy. Total bilirubin was 8.7 mg/dL (direct 4.8 mg/dL), with markedly elevated transaminases (AST 5100 IU/L, ALT 2380 IU/L) and an alkaline phosphatase level of 499 IU/L. Coagulation parameters were significantly deranged, with a prothrombin time of 91 seconds and an international normalized ratio (INR) of 6.53. Serum albumin was 4.8 g/dL. Arterial blood gas analysis revealed alkalemia (pH 7.497), low pCO₂ (17.8 mmHg), and an elevated lactate of 0.98 mmol/L (8.8 mg/dL).

An extensive etiological workup was undertaken. Viral serologies for hepatitis A, B, C, and E were negative. Screening for other infectious etiologies, including malaria and leptospirosis, was unremarkable. Autoimmune markers, including antinuclear antibody and serum immunoglobulin G levels, were within normal limits. Toxicological screening, including serum acetaminophen levels, was non-contributory. Brain imaging could not be done in this patient due to a technical glitch. However, we acknowledge that neuroimaging would have strengthened the evaluation, and this has been our limitation. Ultrasound of the abdomen revealed no significant findings. Doppler ultrasound demonstrated patent portal and hepatic veins, and no features of portal hypertension or splenomegaly were identified. However, detailed portal hemodynamics (portal venous flow velocity, hepatic artery resistance indices) and spleen size assessment were not systematically recorded. This omission is a limitation of our study.

Given the patient’s young age and exclusion of common causes, a metabolic etiology was suspected. Further evaluation revealed a reduced serum ceruloplasmin level (9 mg/dL). Although serum copper levels (92.77 µg/dL) may be unreliable in the setting of ALF, slit-lamp examination demonstrated the presence of Kayser–Fleischer rings. The Kayser–Fleischer rings were identified by slit-lamp examination performed during an ophthalmology consultation and were bilateral, however, detailed characterization regarding density and circumferential extent was not systematically documented at the time of examination. The Leipzig score was 5 (computed in Table 1), which was strongly suggestive of Wilson’s disease presenting as ALF, though not absolutely diagnostic, as confirmatory tests such as 24-hour urinary copper excretion and hepatic copper quantification could not be performed.

**Table 1:** Computed form of Leipzig score.

Parameter	Findings	Points
Kayser–Fleischer rings	Present/absent (not specified)	2
Neurologic symptoms	Not present/not assessed	0
Serum ceruloplasmin	Low	2
Coombs-negative hemolytic anemia	Present	1
Liver copper quantification	Not performed	0
24-hour urinary copper	Not performed	0
ATP7B mutation analysis	Not done/not available	0

Wilson disease-specific chelation therapy could not be initiated because chelating agents were unavailable at our center. Zinc therapy alone was considered unlikely to provide meaningful benefit in the setting of fulminant Wilson disease-associated ALF. The patient was managed with supportive therapy, including intravenous N-acetylcysteine, fresh-frozen plasma for coagulopathy, L-ornithine L-aspartate, and oral lactulose. Despite treatment, serial laboratory parameters demonstrated progressive deterioration. By day 3, transaminase levels declined (AST 402 IU/L, ALT 730 IU/L); however, total bilirubin increased to 12.8 mg/dL (direct 5.34 mg/dL), serum albumin decreased to 3.6 g/dL, and INR remained elevated at 6.5. Arterial blood gas parameters showed partial improvement.

By day 8, there was further clinical and biochemical deterioration, with total bilirubin rising to 17.2 mg/dL (direct 11.9 mg/dL), declining transaminase levels (AST 68 IU/L, ALT 142 IU/L), worsening hypoalbuminemia to 3 g/dL, and severe coagulopathy with failure of clot formation. Biochemical progression over days 1, 3, and 8 is tabulated in Table 2. This pattern of decreasing transaminases in the setting of worsening hyperbilirubinemia and coagulopathy is indicative of massive hepatocellular necrosis and progressive hepatic failure.

**Table 2:** Lab parameters progression across Day 1, 3, and 8.

Parameter	Day 1	Day 3	Day 8
Total bilirubin(mg/dL)	8.7	12.8	17.2
Direct bilirubin(mg/dL)	4.8	5.34	11.9
Aspartate Aminotransferase(IU/L)	5100	402	68
Alanine Aminotransferase(IU/L)	2380	730	142
International Normalized Ratio	6.53	6.5	No clot formation
Albumin(g/dL)	4.8	3.6	3
Alkaline Phosphatase(IU/L)	499	137.1	108
Wbc count (cells/mm^3^)	10 700	14 900	15 300

In view of poor prognostic indicators [New Wilson index: 12, derived as follows: bilirubin (1), INR (4), AST (4), white cell count (3), and albumin (0); total score: 12], the patient was referred to a higher center for urgent liver transplantation. However, due to financial constraints, definitive treatment could not be pursued. On day 10 of hospitalization, the patient’s attendants opted for discharge against medical advice and were subsequently lost to follow-up.

## Discussion

ALF is a rapidly progressive and life-threatening syndrome characterized by acute deterioration of hepatic function, coagulopathy (INR > 1.5), and hepatic encephalopathy occurring within 4 weeks in patients without pre-existing chronic liver disease. It carries a high mortality rate and frequently progresses to multiorgan failure. Emergency liver transplantation remains the only definitive treatment in severe cases^[^5,6^]^.

The etiological spectrum of ALF varies geographically. In developing countries, viral hepatitis and drug-induced liver injury are predominant causes, whereas in Western populations, acetaminophen toxicity, autoimmune disease, and metabolic disorders are more common. Systematic exclusion of these causes is essential before considering rarer etiologies^[^5,6^]^.

In this context, Wilson’s disease is an important but often underrecognized cause of ALF in young individuals. It results from mutations in the ATP7B gene, leading to impaired copper excretion and progressive hepatic accumulation. Excess copper induces oxidative injury, and once hepatic storage capacity is exceeded, free copper enters the circulation, causing systemic toxicity and multiorgan involvement^[^1,2^]^.

Diagnosis of Wilson’s disease in ALF is challenging due to variable biochemical profiles. Low serum ceruloplasmin and Kayser–Fleischer rings provide important diagnostic clues, although serum copper levels may be misleading in acute hepatic failure. Definitive tests, such as 24-hour urinary copper excretion and hepatic copper quantification, may not always be feasible in resource-limited settings, as they were not available in our setting and remain one of the limitations of this study^[^4,6^]^. The Leipzig score is a composite diagnostic system for Wilson’s disease that integrates clinical features, laboratory results, and imaging findings. The cumulative score estimates the probability of disease, with a score ≥4 strongly supporting the diagnosis^[^7^]^. A low alkaline phosphatase-to-bilirubin ratio (<4) has been described as a supportive diagnostic feature of Wilsonian ALF; however, in this case, the ratio on the first day of admission was markedly elevated at 57.3, which does not conform to the classical pattern^[^8^]^.

A key feature observed in this case was the paradoxical decline in aminotransferase levels despite worsening hyperbilirubinemia and coagulopathy. This reflects massive hepatocellular necrosis rather than clinical improvement and is associated with a poor prognosis. The Dhawan prognostic index was 12 in our case, exceeding the mortality threshold of 11 and indicating a poor prognosis consistent with the need for urgent liver transplantation^[^9^]^.

Management of ALF requires intensive care with close monitoring for complications such as cerebral edema and coagulopathy. In non-acetaminophen ALF, N-acetylcysteine has been shown to improve transplant-free survival, particularly in the early stages of encephalopathy. Supportive therapy, including lactulose, fresh frozen plasma, and L-ornithine L-aspartate, is essential. Definitive treatment of Wilson’s disease involves copper chelation therapy and reduction of intestinal copper absorption. However, in fulminant hepatic failure, medical therapy alone is insufficient, and urgent liver transplantation remains the only curative option. Prognosis depends on early diagnosis and timely access to transplantation^[^2,10^]^. However, definitive management could not be pursued because of financial constraints in our case.

## Conclusions

Wilson’s disease should be considered in all young patients presenting with ALF after the exclusion of common etiologies. Early recognition, based on clinical suspicion and supportive findings such as a low ceruloplasmin level and Kayser–Fleischer rings, is crucial. The characteristic biochemical pattern of declining transaminases despite worsening hyperbilirubinemia and coagulopathy reflects progressive hepatocellular necrosis and indicates a poor prognosis. Prompt referral for liver transplantation remains the cornerstone of management in fulminant cases, and delays in diagnosis or limited access to advanced care can significantly impact outcomes.

## Ethical approval

Not applicable.

## Data Availability

Datasets generated during the study shall be made available on request.
